# The utility of biomarkers in hepatocellular carcinoma: review of urine-based ^1^H-NMR studies – what the clinician needs to know

**DOI:** 10.2147/IJGM.S150312

**Published:** 2017-11-27

**Authors:** Caroline R Cartlidge, M R Abellona U, Alzhraa M A Alkhatib, Simon D Taylor-Robinson

**Affiliations:** 1 Department of Surgery and Cancer, Liver Unit, Division of Digestive Health; 2 Department of Surgery and Cancer, Division of Computational and Systems Medicine, Faculty of Medicine, Imperial College London, London, UK

**Keywords:** hepatocellular carcinoma, biomarkers, metabonomics, urine, proton nuclear magnetic resonance spectroscopy, ^1^H-NMR

## Abstract

Hepatocellular carcinoma (HCC) is the fifth most common malignancy, the third most common cause of cancer death, and the most common primary liver cancer. Overall, there is a need for more reliable biomarkers for HCC, as those currently available lack sensitivity and specificity. For example, the current gold-standard biomarker, serum alpha-fetoprotein, has a sensitivity of roughly only 70%. Cancer cells have different characteristic metabolic signatures in biofluids, compared to healthy cells; therefore, metabolite analysis in blood or urine should lead to the detection of suitable candidates for the detection of HCC. With the advent of metabonomics, this has increased the potential for new biomarker discovery. In this article, we look at approaches used to identify biomarkers of HCC using proton nuclear magnetic resonance (^1^H-NMR) spectroscopy of urine samples. The various multivariate statistical analysis techniques used are explained, and the process of biomarker identification is discussed, with a view to simplifying the knowledge base for the average clinician.

## Background

Globally, hepatocellular carcinoma (HCC) is the fifth most common malignancy in men and the seventh in women.^1^ HCC is up to four times more common in men and is the most common primary liver cancer,^2^ with most cases occurring in sub-Saharan Africa and East Asia, due to the high prevalence of hepatitis B virus (HBV).^3^–^5^ Roughly 80% of HCCs occur on a background of cirrhosis.^6^ The mortality rate is almost identical to the incidence, which is increasing.^7^ However, in Taiwan, HBV vaccination programs have reduced incidence rates.^8^

HCC is the second leading cause of cancer death, with over half a million cases diagnosed per year,^1^ although data quality varies worldwide. Prognosis is poor, with 5-year survival <5% due to late presentation, as HCC is often asymptomatic in the early stages.^9^ However, HCC is potentially curable, following the defined treatment algorithm,^10^ if detected early, but liver transplantation must conform to strict criteria used to assess suitability of patients with cirrhosis and HCC, such as the Milan criteria.^11^

In terms of the importance of this review topic, urine-based nuclear magnetic resonance studies are considered valuable methods of analyzing large quantities of data and have a wide variety of uses. Most notably of these uses is the detection of biomarkers, which have increasingly important roles in disease discovery and monitoring. Furthermore, there is the advantage of urine-based tests being noninvasive, compared to blood tests, which can cause discomfort or harm. Greater understanding of this topic offers further insight into disease development, prognosis, and pathogenesis. This is done through achieving metabolic and spectral fingerprints of urine, which allow for identification of relevant signals, that differ in ill health. These signals are ultimately processed using multivariate analysis techniques to identify biomarkers for disease, which aim to detect diseases, such as HCC, earlier to resultantly improve prognosis.

## HCC risk factors

Chronic hepatitis and cirrhosis of any cause accounts for 70–90% cases of HCC occurring on a background of chronic liver disease.^6^ Human immunodeficiency virus co-infection with either HBV or hepatitis C virus (HCV) leads to quicker progression to cirrhosis and HCC.^12^ Of note, 80% of HCC cases occur in Eastern Asia and sub-Saharan Africa, where the main risk factor remains to be chronic HBV. HCC incidence increases when there are higher levels of HBV DNA and with a longer duration of infection.^13^ HBV has two main ways of causing HCC:

1. HBV causes cirrhosis from chronic inflammation of the liver, predisposing to HCC and
2. HBV is directly oncogenic, whereby the virus integrates into the host genome, causing carcinogenic mutations.^14^

By contrast, HCV is the main risk factor for HCC in Europe, North America, and Japan. Roughly 3–5% of individuals with HCV cirrhosis develop HCC per year.^15^ Acute infection leads to chronic hepatitis and then cirrhosis, thereby leading to the risk of HCC. However, fortunately antiviral therapy may reduce this risk.^16^ Alcohol and tobacco abuse are further risk factors for HCC. Prolonged, heavy drinking (40–60 g alcohol per day) is a well-established risk factor for HCC, independently and in combination with HCV.^17^ Alcoholic cirrhosis has a role in up to 35% of all HCCs.^18^,^19^ Tobacco independently increases the risk of HCC in cirrhotic patients with a dose-dependent effect.^20^ Furthermore, an increase in the number of cases of non-alcoholic fatty liver disease in more developed countries due to a more obesogenic environment is an emerging risk of HCC.^21^,^22^ However, estimates of risk are unclear. HCC has been found to be twice as likely to develop in diabetic patients compared to non-diabetics.^23^,^24^

Aflatoxin is a carcinogenic mycotoxin produced from certain species of fungi such as *Aspergillus flavus*, which colonizes food crops, most heavily impacting developing countries.^25^ HCC occurs through chronic low-level ingestion from dietary aflatoxin contamination,^26^ as aflatoxin is metabolized in the liver, allowing for its toxic effects to be exerted. Tumor suppressor gene *p53* becomes mutated at the third position of codon 249, leading to the development of HCC.^27^ Aflatoxin B1 is the most toxic and prevalent type, further described as the most potent known experimental carcinogen.^28^ There is up to a 30 times increased risk of HCC when individuals are also infected with HBV, suggesting that HBV and aflatoxin promote HCC development synergistically.^29^

## Currently available biomarker: alpha-fetoprotein (AFP)

The current gold-standard and only established noninvasive diagnostic marker for HCC is serum AFP, but this fails in diagnostic performance ability, making it unreliable.^30^ The American Association for the Study of Liver Diseases (AASLD) describes the relationship of AFP to HCC as lacking “adequate sensitivity and specificity for effective surveillance and diagnosis”.^10^ Serum AFP level and 6-monthly ultrasound (US) scans are used for surveillance and diagnosis of HCC.^5^ However, these are often unsuitable for resource-limited settings as imaging is often expensive or unavailable. Furthermore, sensitivity and specificity are known to be poor. Sensitivity of US to discover malignant liver lesions and characteristic changes in vascularization is often operator dependent. AFP alone has been reported to detect ~70% of HCC cases as it is only produced by about two-thirds of cases.^30^ Area under receiver operating characteristic curve values are seen to vary between 0.60 and 0.70,^31^ which highlights the need for a more effective diagnostic biomarker.

However, AFP is not the only available biomarker for HCC. There are many others, including cell surface heparan sulfate proteoglycan glypican 3, circulating microRNAs (miRNAs), and abnormal coagulation protein des-gamma carboxyprothrombin, also known as PIVKA-II (protein induced by vitamin K absence or antagonist II). Despite having great potential, the sensitivity and specificity of these have been disappointing and consequently none are routinely used in clinical practice around the world.

## Metabonomics and its utility in HCC

To study disease biomarkers, metabonomics can be used. It has been defined by Nicholson et al as “the quantitative measurement of the dynamic multiparametric metabolic response of living systems to pathophysiological stimuli or genetic modification”.^32^ This differs from metabolomics which identifies and analyzes all metabolites in a system,^33^,^34^ although terms are often used interchangeably. Metabonomics is useful to investigate HCC, because in terms of molecular pathogenesis, there are 30–40 mutations per tumor,^35^ and according to the Warburg hypothesis, tumors have altered metabolism compared to healthy cells.^36^,^37^ More specifically, there are variations in mitochondrial respiration as malignant tissue consumes more glucose. Consequently, the key focus is on metabolites associated with energy, altered lipid metabolism, and membrane turnover for identification of potential HCC biomarkers.^38^ Once identified, in the future, these biomarkers would have the potential to be detected in urine using a simple dipstick test, with the aim for it to be cheap and readily available at the point of care, to help identify the presence of HCC.

## Why use urine in metabonomic studies to identify biomarkers?

Urine is a stable and convenient biofluid for metabonomics because collection is noninvasive and easily accessible. Urine can be used for widespread screening and surveillance through a simple dipstick test, suited to the developing world where cost and access to imaging techniques are more problematic. Consequently, work is being done involving urine samples to identify and validate metabolites for HCC that could act as potential biomarkers for accurate detection of the disease.

## Proton nuclear magnetic resonance (^1^H-NMR) spectroscopy background

The NMR phenomenon can be applied as magnetic resonance imaging (MRI) to provide anatomical structural details and study tissue metabolism in vivo. However, for the analysis of urine samples, ^1^H-NMR spectroscopy is used, which is a noninvasive analytical chemistry technique and is used to determine protein structure and chemical composition of biofluids. A familiarity and basic knowledge of ^1^H-NMR theory for readers is assumed.

Furthermore, ^1^H-NMR spectroscopy has been used for metabolic phenotyping since the 1980s. Mass spectrometry (MS) is more sensitive, but ^1^H-NMR is more robust and versatile, with very high reproducibility when there is consistent sample preparation and setup.^39^ The use of ^1^H-NMR spectroscopy for biomarker discovery has the advantage of simpler sample preparation and more reproducible results. However, it is not as sensitive as liquid chromatography mass spectrometry (LCMS) and it has the challenge of signal overlap leading to difficulty in making assignments. While LCMS offers greater sensitivity, it is more laborious to set up and results depend on individual experimental conditions which make metabolite assignments and data analysis more challenging. ^1^H-NMR is non-destructive and, therefore, precious samples can be reused for biomarker determination, unlike in MS. Consequently, ^1^H-NMR spectroscopy is deemed to be a well-suited technique for analysis of samples in metabonomic studies. As neither technique can detect all chemical compounds present in a biofluid sample, the two approaches should be considered complementary in the discovery of biomarkers. This review will focus on the workflow of biomarker discovery using ^1^H-NMR.

## Chemical shift and metabolite interpretation

The chemical shift phenomenon refers to the differences in electron density surrounding nuclei depending on the local chemical environment within molecules which causes variation in the opposing magnetic field.^40^ This is integral in ^1^H-NMR spectroscopy for metabolite interpretation and biomarker identification in metabonomics. Chemical shift is calculated by determining the difference in resonance frequency of the nucleus, against a standard. 3-(Trimethylsilyl)-2,2′,3,3′-tetradeuteropropionic acid (TSP) is most commonly used as an internal chemical shift reference in metabonomics for reasons listed in Table 1.^41^,^42^ However, TSP can be affected by protein in urine, causing the peak to become smaller and wider, as protein binds to TSP. Peak position is indicative of the chemical environment, whereas peak area is proportional to the number of nuclei with the same chemical environment generating the signal and the overall concentration of the compound in the sample.

Chemical shift (d) is expressed on the horizontal scale, where TSP is assigned to zero parts per million (ppm) to serve as the reference peak. ppm measures frequency signals instead of Hertz, on the x-axis, as it more simply represents frequency and aids comparison of spectra from spectrometers with different magnetic strength. Moreover, ppm is used when assigning metabolite identity to determine what is present in a sample.

## NMR applications within hepatology

NMR spectroscopy has several versatile research applications in hepatology, as the liver has a variety of metabolic and detoxification functions to be assessed and interpreted. These include using markers to assess the functional capacity of the cirrhotic liver,^43^ grading liver disease in hepatitis C,^44^ and identifying biomarkers of cholangiocarcinoma in bile.^45^ Advancements are constantly being developed to improve NMR performance, such as the ongoing invention of the “microcoil” for enhanced resolution of in vivo MRI.^46^ Metabonomic biomarker discovery is an important application of ^1^H-NMR within hepatology, as many papers are now focused on identifying biomarkers in HCC. However, it must be noted that ^1^H-NMR is not intended to be a replacement for imaging but is to be used as a complementary technique.

## In vitro urine ^1^H-NMR spectra

^1^H-NMR spectroscopy of urine aims to acquire high-resolution spectra, to determine composition by identifying metabolites, without prior structural knowledge. Sample randomization prior to analysis is important to avoid bias from operator differences or conditions.^47^ The ^1^H-NMR urine spectrum is generally composed of low molecular weight metabolites, forming thousands of sharp resonances.^48^ Urine has a high water content; therefore, water suppression achieved by constant irradiation which saturated the resonance of water is key to reduce interference for optimum detection of metabolites. ^1^H-NMR offers high reproducibility without compensating throughput; therefore, spectra of the same urine sample should be superimposable, making it a suitable mode of analysis for detecting potential biomarkers of HCC.

High-quality samples are required for successful metabonomic studies, but quality may have varied between samples in different studies. Similarly, in comparison, some samples will have been left in the NMR spectrometer for longer than others. Although, like the logistical delay between collecting and analyzing samples, this is not expected to impact upon the urinary ^1^H-NMR profile, as urine is metabolically stable unless contaminated.^49^ However, several factors influence urinary metabolic profiles.

## Factors affecting ^1^H-NMR urine spectra

A variety of factors can affect the utility of biomarkers in HCC due to variations in ^1^H-NMR urine spectra which impacts upon the identification of metabolites as potential biomarkers. However, given the vast assortment of metabolites found in urine, wide variations in human gene-environment exposure can make disease biomarkers even more difficult to identify.^50^

Diet affects the gut microbiome and is the biggest contributor altering urinary metabolites. This has been demonstrated by studies considering dietary variation and metabolic profiles of urine, showing diet can be a confounding factor in disease biomarker studies. For example, variations in urinary metabolites are seen between vegetarian diets and in meat eaters, thereby suggesting differences in urine metabolic signatures, based on diet. Diets including meat have shown increased concentrations of metabolites, such as creatinine, carnitine, acetylcarnitine, and trimethylamine-*N*-oxide (TMAO). In contrast, those on vegetarian diets have higher levels of *p*-hydroxy phenylacetate, suggesting that alterations in the microbiome and diet contribute to urinary metabolic profile.^42^ Moreover, urine samples are more affected by changes in the diet than serum samples.^51^

Drugs such as non-steroidal anti-inflammatory drugs can affect the metabolic composition of urine.^52^ Most notably, antibiotics affect the gut microbiome, which may alter urinary metabolites.^53^ Similarly, paracetamol metabolites in urine heavily influence analysis and therefore they could be justifiably excluded from further analysis if discovered.^54^ Dietary supplements, herbal remedies, and over-the-counter medications can in cases be found in urine, but with difficulty to determine the identity of the compound.^47^ Overall, interference by drug metabolites can cause changes in urine composition to be more difficult to identify or missed altogether. Likewise, urinary changes have been identified due to diabetes.^55^,^56^ If diabetes is detected in urine spectra by high glucose, the peak should be removed from analysis if possible. The whole sample should be justifiably excluded from further analysis because if the peak is so strong it biases the model.

The degree of physical activity is directly associated with metabolism, whereby different levels of exercise have short- and long-term effects. For example, urine lactate increases after exercise,^57^ among a variety of other changes in urinary metabolites. ^1^H-NMR has the potential to differentiate between urine taken before and after exercise, according to metabolic profile.^58^ Genetic factors contribute to the high variability of the human urine metabolome due to inter-individual differences.^47^ Age has also been suggested to play a role in urine metabolite levels as ^1^H-NMR spectra have been found to distinguish between those of young and old.^59^ Furthermore, time of sample collection is important to monitor and keep consistent, as some studies have seen diurnal variation in all urine samples.^60^

## Metabolites found to be discriminatory for HCC

Hormone profiles differ between men and women and significant differences in steroid metabolite excretion profiles have been identified, among other metabolite variations affecting ^1^H-NMR urine spectra.^61^ Moreover, female hormones are known to be protective against HCC, whereas male hormones, like testosterone, have been shown to correlate with HCC risk.^62^ Evidence suggests this could relate to interleukin-6 (IL-6), which is a mediator synthesized by Kupffer cells that promotes HCC but is inhibited by estrogens. In mouse models, when IL-6 was ablated, there were no longer gender differences in hepatocarcinogenesis.^63^

Altered composition of urinary metabolites in HCC vs controls has been previously confirmed by ^1^H-NMR studies,^38^,^49^,^54^,^64^ whereby reportedly discriminatory candidate biomarkers are described in Table 2. However, in most cases, there is also a general trend in the difference between HCC and cirrhotic patients, but not often shown to be significant. Many Chinese studies^65^–^69^ have also been carried out comparing HCC with healthy controls by considering HCC urinary biomarkers using MS, but there is a need for more large-scale African studies, where HCC is an issue of equivalent magnitude. Each study highlights the need for further validation studies to discriminate between metabolic phenotypes of disease states alone, such as HCC, HBV, and cirrhosis, as most HCCs occur on the background of cirrhosis; therefore, metabolic differences in chronic liver disease also need to be identified. Creatinine,^49^,^54^,^64^,^65^ hippurate,^49^,^64^ citrate,^64^,^67^ and carnitine^49^,^54^ are the main metabolites corroboratively reported to be discriminant in HCC based on independent studies. For more information beyond the scope of this review, the uses and derivation of notable metabolites in HCC are detailed in a thorough review by Kimhofer et al.^70^

Creatinine is the breakdown product of creatine phosphate in muscle; therefore, reduced creatinine may be found because of decreased muscle mass, linked to cancer cachexia. Renal impairment causes increased serum creatinine and, therefore, it is important to check for differences in renal function between disease classes. Dietary animal protein increases creatinine levels,^38^ but it is rare for sub-Saharan individuals to consume much meat due to economic constraints. Diet in sub-Saharan Africa mostly consists of vegetables and crops, such as yam, and fish in coastal regions. Overall, the reduction of creatinine in previous publications is likely to be due to the diverse effects of the tumor on physiology.^49^,^54^,^64^,^65^

Benzoate is formed by the metabolism of gut microbes from dietary aromatic compounds due to reduced hepatic function in HCC.^71^,^72^ Consequently, hippurate, an acyl glycine formed by conjugation of benzoate and glycine in liver and kidney mitochondria,^73^ may act as a surrogate marker of hepatic function. Hippurate levels have been found to be reduced in previous studies^38^,^49^,^64^ due to less efficient benzoate conjugation, possibly from dysbiosis of microbiota, because of the disease.

Previous publications have also reported a reduction of citrate in HCC.^38^,^64^,^67^ Citrate is an intermediate in the Krebs cycle and, therefore, downregulation of citrate follows the Warburg effect relating to alterations in mitochondrial aerobic respiration of tumor cells.^36^,^37^ However, this is not specific for HCC. Citrate concentration has been shown to be higher in women, which is postulated to relate to estrogen levels,^74^,^75^ but more research is required.

Carnitine is mainly derived from the diet and has an essential role in mitochondrial metabolic pathways as a product of tumor respiration.^64^ Significantly raised urinary carnitine levels have been found in HCC,^49^,^54^ whereas in healthy individuals <5% is usually excreted.^76^ Carnitine overproduction may result from rapid tumor growth, fueled by increased mitochondrial activity. Carnitine is important for energy metabolism, specifically as a cofactor to transport fatty acids from cytosol to mitochondria for the initial phase of beta-oxidation.^77^

## Process of biomarker determination

**Figure 1:**
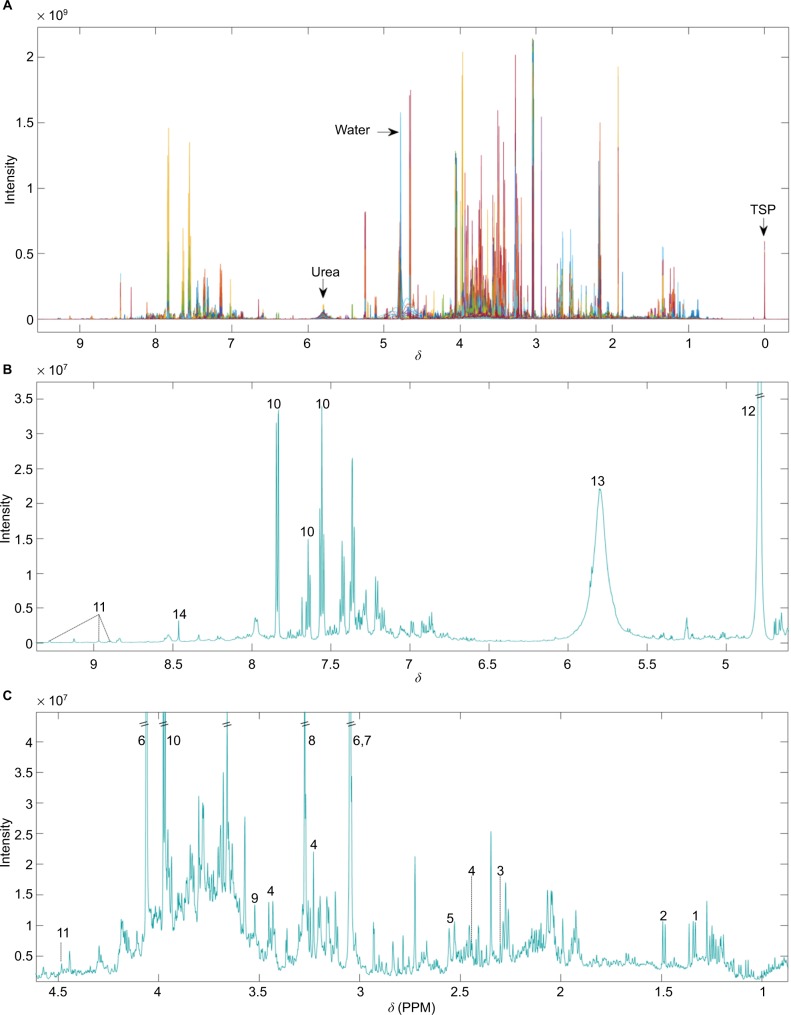
(**A**) Unedited ^1^H-NMR spectra example. Different colors correspond to individual samples overlaid. (**B**) Median spectra of unedited data for ppm values ranging from above 4.5 to below 9.5. (**C**) Median spectra of unedited data for ppm values ranging from 1 to 4.5. Numbers indicate the following commonly reported metabolites – 1: lactate; 2: alanine; 3: acetoacetate; 4: carnitine; 5: citrate; 6: creatinine; 7: creatine; 8: TMAO; 9: glycine; 10: hippurate; 11: 1-methylnicotinamide; 12: water; 13: urea; 14: formate. **Abbreviations:** ^1^H-NMR, proton nuclear magnetic resonance; *δ*, chemical shift; PPM, parts per million; TMAO, trimethylamine-N-oxide; TSP, 3-(trimethylsilyl)-2,2′,3,3′-tetradeuteropropionic acid.

There is quite a challenging process involved in biomarker determination, involving several important steps. ^1^H-NMR spectral data must be acquired from urine samples following standardized protocols conforming to experimental protocols by Dona et al^48^ and then must be matched with associated demographic and anonymized clinical data. Pre-processing is carried out on the unedited spectra (Figure 1), involving the removal of the uninformative TSP, water and urea regions, alignment, and normalization.

**Figure 2:**
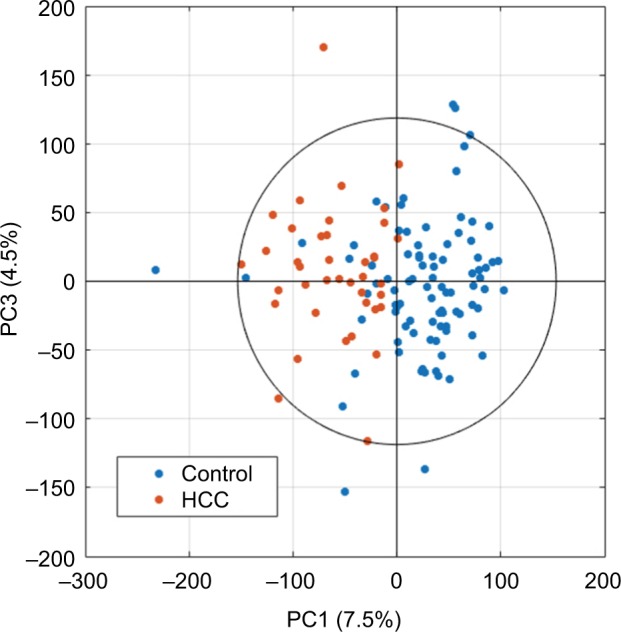
Example of univariance scaled PCA scores plot after data processing using the first and third principal components. **Abbreviations:** PCA, principal component analysis; HCC, hepatocellular carcinoma; PC, principal component.

After editing of spectra, scaled principal component analysis (PCA) score plots (Figure 2) can be generated to summarize all samples, with potential to identify trends in data, and remove outliers determined by the Hotelling T^2^ confidence interval,^78^ if well-reasoned, such as interference from drug metabolites or glucose. PCA is an unsupervised method involving principal components that are linearly uncorrelated coordinates used to express the greatest variance within a data set in decreasing orthogonal fashion. Component scores describe variations between samples.^79^

**Figure 3:**
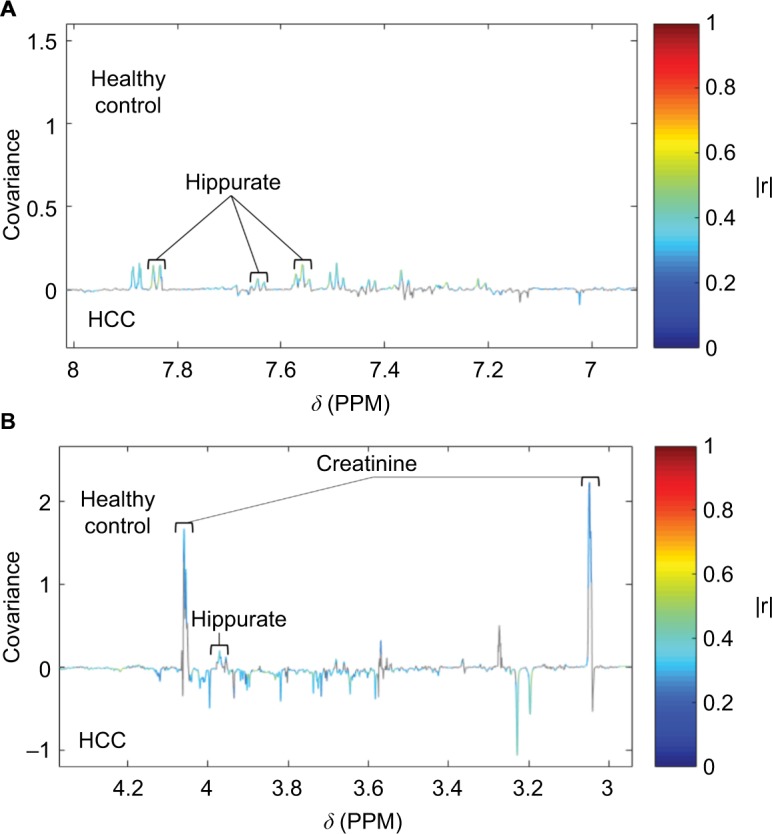
Loading plot from an OPLS-DA model built using HCC and healthy control as disease classes pairwise. **Notes:** (**A**) Loading plot ranging from 7 to 8 ppm, with labelled peaks signifying hippurate metabolites. (**B**) Loading plot ranging from 3 to 4.2 ppm, with labelled peaks signifying creatinine and hippurate metabolites. Significant signals (pFDR<0.05) are shown in colors corresponding to the correlation coefficient (|r|). Hippurate and creatinine metabolites are labeled. **Abbreviations:** HCC, hepatocellular carcinoma; OPLS-DA, orthogonal partial least squares discriminant analysis; pFDR, false discovery rate; *δ*, chemical shift; PPM, parts per million.

Orthogonal partial least-squares discriminant analysis is a supervised method used to generate loadings plots. These show statistical deviations in response to the difference between the variable of interest, used to help determine peaks that significantly differ, such as the classification of different patient groups or certain clinical measurements (Figure 3).

**Figure 4:**
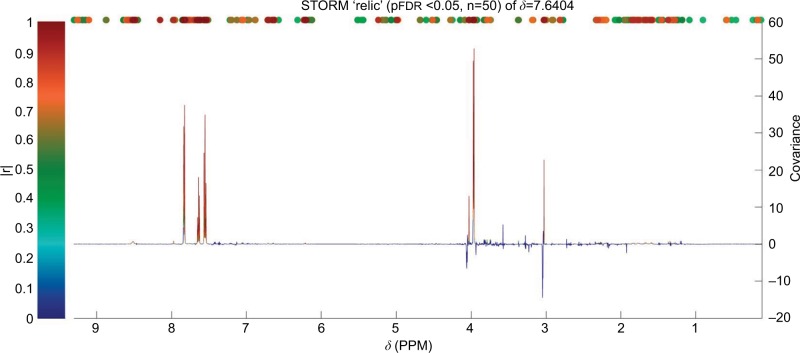
Identification of peaks from the same compound (hippurate) using STORM.^50^ Significant signals (pFDR<0.05) are shown in colors corresponding to the correlation coefficient (|r|). **Abbreviations:** STORM, subset optimization by reference matching; *δ*, chemical shift; PPM, parts per million; pFDR, false discovery rate.

Statistical total correlation spectroscopy (STOCSY) is a technique developed by Cloarec et al to help determine the chemical structure of the molecule responsible for a peak of interest.^80^ Signals are identified from specific metabolites as well as associated signals involved in the same pathway. This is done by inputting ppm value of a signal and then the correlation between resonances is calculated. Posma et al’s development of subset optimization by reference matching (STORM)^50^ offers improved visualization of peaks (Figure 4), compared to STOCSY, as well as the ability to select subsets of ^1^H-NMR spectra. This optimizes the statistical approach to identify and assign metabolites.

However, multiple testing is prone to false positives; therefore, to help determine the true significance of signals, Bonferroni–Hochberg^81^ method of *p*-value correction for multiple testing is an important step to include to ensure significant results are not due to chance.^82^

Assignment of metabolites is then done by comparing spectral signals to published literature and databases, such as the Human Metabolome Database (HMDB), or by searching internal reference databases according to metabolite name or ppm value of ^1^H-NMR signal. Furthermore, experiments involving spiked-in quantities of putatively assigned metabolites act as the gold standard, forming the basis of biomarker discovery.

**Figure 5:**
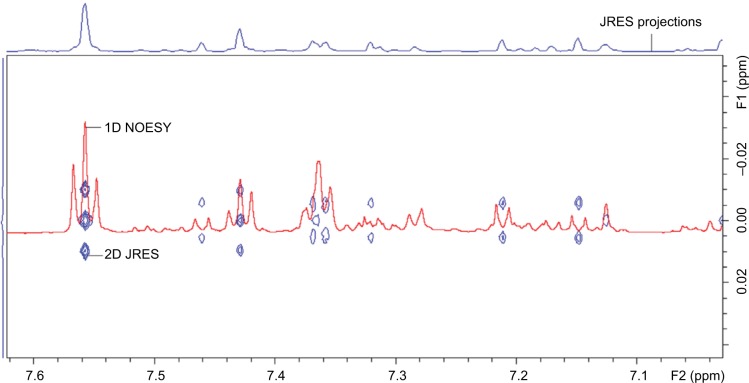
Example of 2D JRES image (blue) with 1D NOESY spectra overlaid (red) used to verify multiplicity. **Notes:** F1 axis shows the proton-proton coupling. F2 axis shows the chemical shift. **Abbreviations:** JRES, J-resolved spectroscopy; 2D, two-dimensional; 1D, one-dimensional; NOESY, nuclear Overhauser effect spectroscopy; ppm, parts per million.

Furthermore, two-dimensional (2D) JRES (J-resolved spectroscopy)^83^ ^1^H-NMR data is useful alongside the overlay of one-dimensional (1D) spectra to verify multiplicity of peak signals (Figure 5). In general, 2D NMR is mainly used to provide additional detail of complicated molecular structures, through increased scan number.^84^

One limitation of publications so far is that many compounds are yet to be identified due to peak overlap being problematic for assignment and metabolites not documented in databases. 2D NMR data is underused, such as correlation spectroscopy^85^ and total correlation spectroscopy.^86^ However, they can aid confirmation of metabolite assignments by providing additional information about the relationship between peaks, to help with overlap. Similarly, more studies including spike-in experiments are required to confirm metabolite identity, which involves analyzing authentic compounds added to samples, to observe the peaks produced. However, both 1D and 2D NMR can sometimes be inconclusive, making metabolite assignment more challenging. For instance, in cases where spectra are of poor quality, insufficient intensity, or have resonance overlap.

## Conclusion

This review is based on the observation of an unmet medical need for an effective, reliable, and affordable diagnostic HCC test. HCC is an important issue due to its poor prognosis and late diagnosis.^87^ Consequently, in some parts of the world, HCC is the leading cause of death for those under 40 years old.^5^ Urine is a suitable biofluid for ^1^H-NMR spectroscopy to detect significant markers of HCC, as urine samples are easily accessible and acceptable to patients. Similarly, the development of a simple urine dipstick test for HCC based on the diverse effects of HCC tumorigenesis on metabolic pathways would be inexpensive and convenient for use in resource-deprived areas, where HCC incidence is highest.^3^,^4^ ^1^H-NMR spectroscopy is useful in the development of a candidate biomarker panel. Such dipstick technology is a future line of research but has not yet been effected. Metabonomic studies and biomarkers are now even more essential to continue improving as we need to be looking beyond the genome to make more progress toward facilitating the diagnosis, treatment, and cure of HCC.

## Future directions

Overall, studies so far suggest that urinary diagnostics are possible, and metabolites associated with energy, altered lipid metabolism, and markers of cell membrane turnover are probably discriminatory for HCC. However, larger studies are required, involving multiple sample populations, including different geographical locations, genetics, and etiologies of HCC. Furthermore, it is important to pay attention to confounding factors of ^1^H-NMR in studies, such as diet, smoking, and drugs. The inclusion of more women is also key to identifying and confirming the significant metabolites for a diagnostic panel of HCC biomarkers. Further independent validation studies of new sample sets are needed, with comprehensive clinical data, including AFP for comparison of diagnostic performance against any potential new biomarkers identified for HCC. Ultimately, this should lead to the development of a novel pregnancy test–style urine dipstick test for HCC, which is theoretically plausible, and possible in the future, after further research and development. Biomarkers in HCC could have a massive medical impact in alleviating disease burden through low cost, earlier detection, and before the cancer stage is too late for curative treatment.

## Figures and Tables

**Table 1:** Reasons for using TSP as a reference standard during ^1^H-NMR spectroscopy

• Reference peak with chemical shift frequency is far from that of signals from metabolites of interest
• Resonance of TSP does not overlap with sample components
• TSP is chemically stable
• TSP does not interact with sample components
• Shifts from TSP are precisely characteristic of certain nuclei within a metabolite and are very sensitive to environmental changes such as pH and temperature, although there is minimal variation when prepared accurately
• TSP dissolves well in aqueous solvents used in metabonomics, unlike tetramethylsilane (TMS), another commonly used reference standard in ^1^H-NMR
• TSP generates a single signal peak, unlike TMS which can give more, thereby obstructing the metabolic profile of the spectra
• TSP can be used as a concentration reference to calculate concentration of metabolites

**Note:** Data from Holzgrabe et al^41^ and Stella et al.^42^

**Abbreviations:** TSP, 3-(trimethylsilyl)-2,2′,3,3′-tetradeuteropropionic acid; ^1^H-NMR, proton nuclear magnetic resonance.

**Table 2:** Previously published studies aiming to identify HCC biomarkers through analysis of urine by ^1^H-NMR spectroscopy

Studies	Year	Country	Main HCC etiology	Significant metabolites discriminatory for HCC vs controls	↓/↑
Shariff et al^54^	2010	Nigeria	HBV	Creatinine	↓
				Carnitine	↑
Shariff et al^64^	2011	Egypt	HCV	Glycine, TMAO, hippurate, citrate	↓
				Creatine	↑
Ladep et al^38^	2014	Nigeria	HBV	Hippurate, TMAO, pyruvate, citrate	↓
				Acetylcarnitine, dimethylglycine, carnitine, indole-3-acetate, creatine, methionine, unknown spectral signal putatively assigned to N-acetylated amino acid, 2-oxoglutarate	↑
Cox et al^49^	2016	Bangladesh	HBV	Creatinine, hippurate, TMAO	↓
				Carnitine	↑

**Notes:** Statistically significant metabolites discriminatory for HCC vs controls are shown and arrows indicate whether increased or decreased in HCC.

**Abbreviations:** HCC, hepatocellular carcinoma; HBV, hepatitis B virus; HCV, hepatitis C virus; TMAO, trimethylamine-N-oxide; ^1^H-NMR, proton nuclear magnetic resonance.
